# Tape-Fixation for the Treatment of a Dorsal Boss in Lisfranc Joints of an Elderly Patient

**DOI:** 10.7759/cureus.70265

**Published:** 2024-09-26

**Authors:** Takeomi Nakamura, Ryoma Yoshida, Isaku Saku

**Affiliations:** 1 Orthopaedics, Tokyo Metropolitan Hiroo Hospital, Tokyo, JPN; 2 Department of Orthopaedics and Traumatology, JR Tokyo General Hospital, Tokyo, JPN; 3 Department of Orthopaedics and Traumatology, Yaizu City Hospital, Shizuoka, JPN

**Keywords:** surgery in elderly, quality of life (qol), bone spur, dorsal boss, lisfranc osteoarthritis, osteoarthritis of the foot, instability of tmt joint, bc internal brace, semi-rigid fixation, tape augmentation

## Abstract

A dorsal boss, also known as a tarsal boss, is a bony prominence often associated with osteoarthritis (OA) of the tarsometatarsal (TMT) joints, leading to significant pain and a reduced quality of life (QOL) in elderly individuals. This condition frequently forces patients to abandon recreational activities and is typically resistant to conservative treatments. This report details a successful surgical intervention in an 83-year-old female patient with a dorsal boss and OA of the TMT joint, which involved osteophyte excision and semi-rigid fixation using ligament tape with an absorbable screw (Arthrex, Inc., Florida, USA). Post-surgery, the patient, who had experienced pain and deformity in the dorsal region of her right foot, showed significant improvement and returned to playing golf three months later.

This case underscores the significance of considering a semi-rigid, flexible dorsal fixation approach in elderly patients with dorsal bosses and associated joint instability while preserving joint surfaces and facilitating early reintegration into society. The patient’s favorable outcome highlights the potential advantages of this surgical method, particularly in managing dorsal boss cases that are resistant to conservative treatment.

## Introduction

Maintaining the quality of life (QOL) and independence of elderly individuals in modern society is vital. Therefore, physicians must focus on specific anatomical areas to ensure the health and well-being of the feet in this population. Osteoarthritis (OA) of the tarsometatarsal (TMT) joint often results in pain and a decline in QOL [1], leading many individuals to forgo recreational activities such as sports.

As OA progresses, osteophytes can increasingly develop on the dorsal aspect of the joint. This can sometimes be accompanied by pain, leading to what is commonly referred to as a dorsal boss of the foot [2]. A symptomatic dorsal boss may not respond to conservative treatment [2]. However, information in published studies describing surgical resection of the dorsal exostosis of the second and third metatarsocuneiform joints, as well as the management of patients with neuritis of the intermediate dorsal cutaneous nerve, is lacking [3].

To the best of our knowledge, no existing literature has addressed the use of semi-rigid fixation with an artificial ligament for this type of arthritis. We believe our case will be the first to report mid-term outcomes of tape ligament augmentation for this condition. We aim for this work to provide valuable insights and enhance the current body of evidence.

## Case presentation

**Figure 1:**
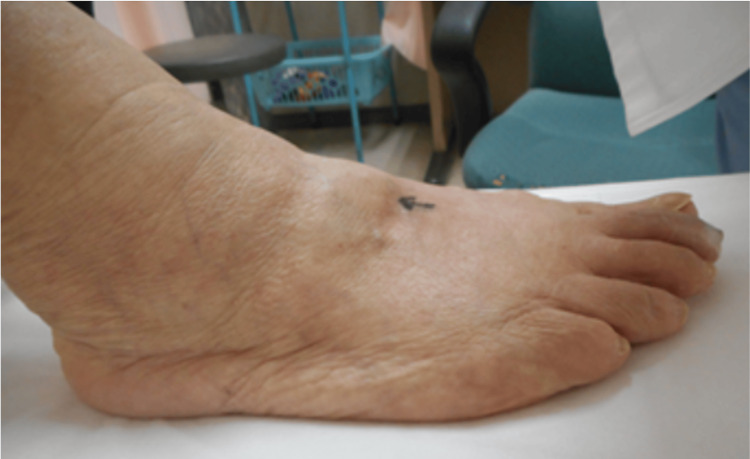
Clinical view demonstrating the dorsal boss, indicated by an arrow on the skin The patient reported experiencing pain while wearing shoes and during ambulation.

**Figure 2:**
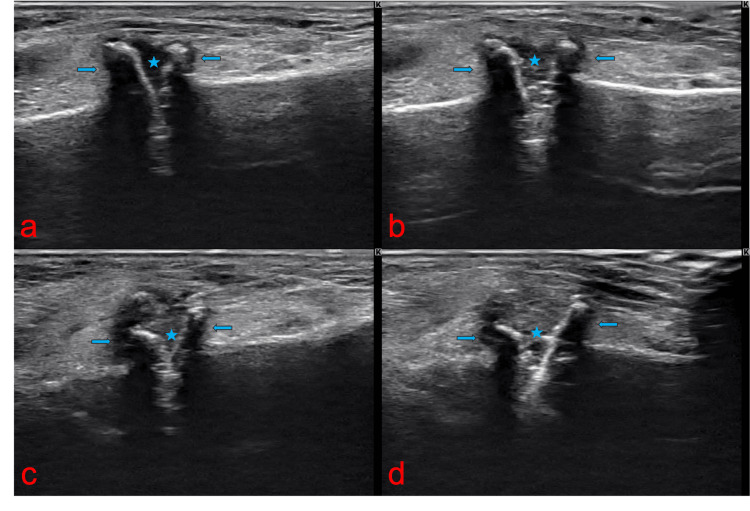
Ultrasound investigation The stars indicate the joint spaces, while the arrows highlight the osteophytes. (a) Neutral position of the second TMT joint showing joint space opening dorsally; (b) stress study in plantar flexion of the second TMT joint, revealing a wider joint space compared to the neutral position; (c) neutral position of the third TMT joint, similarly exhibiting joint space opening dorsally; (d) stress study in plantar flexion of the third TMT joint, demonstrating a wider joint space than in the neutral position. Following the excision of the osteophytes, there was significant concern about the potential for further joint instability.

**Figure 3:**
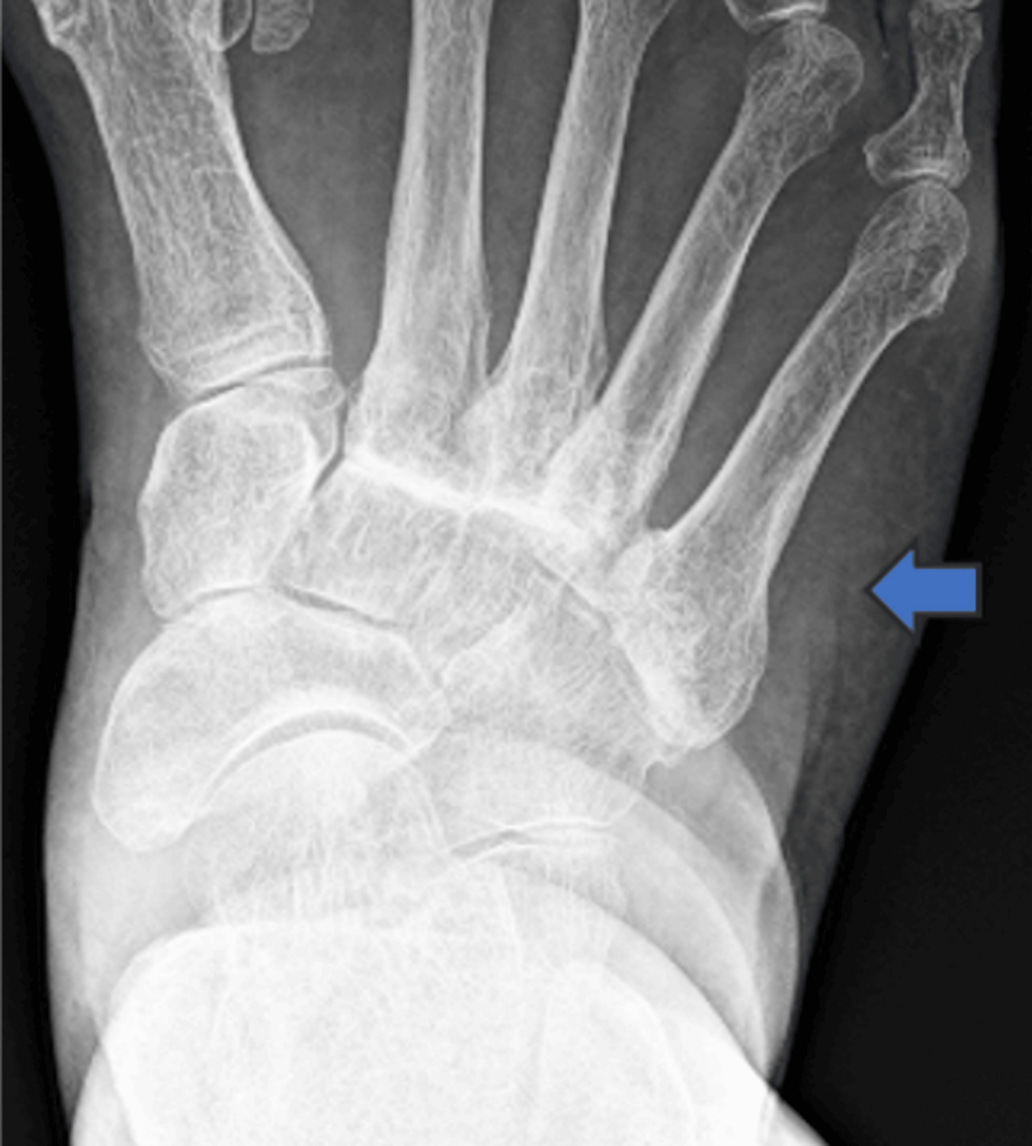
Preoperative X-ray The arrow indicates degenerative changes in the TMT joints, including hardening of the joint lines. TMT: tarsometatarsal

**Figure 4:**
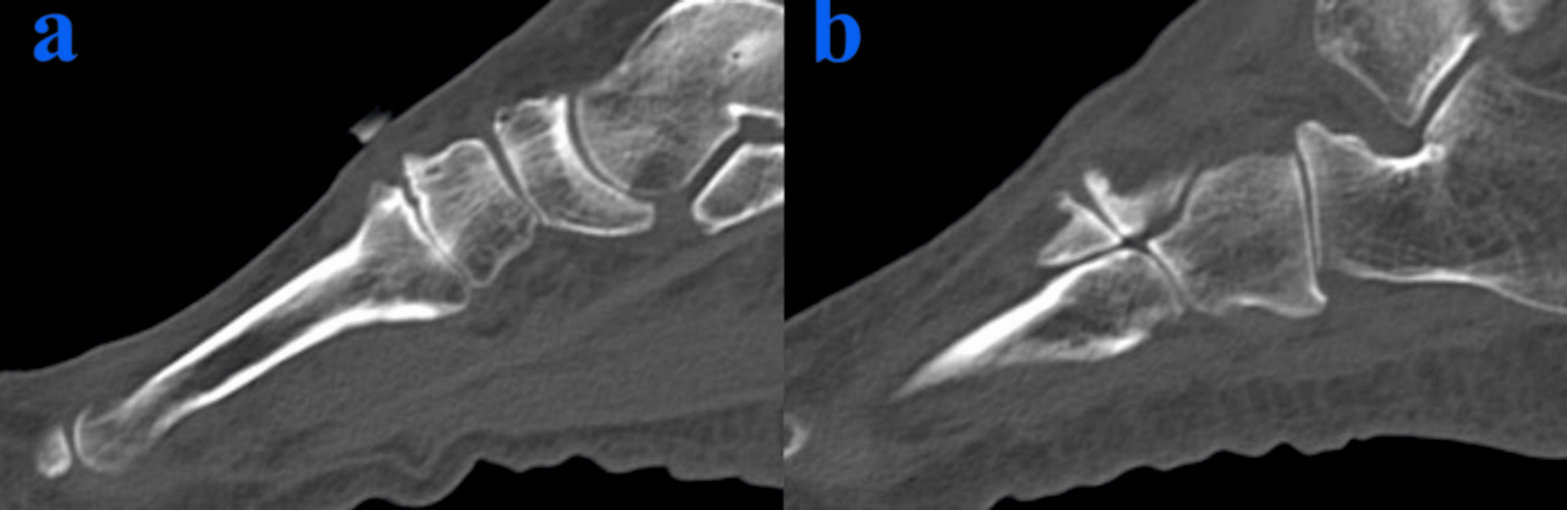
Preoperative CT scan (a) Second TMT joint; (b) third TMT joint TMT: tarsometatarsal The sagittal view of the images demonstrates osteophyte formation, commonly referred to as a dorsal boss. No significant articular changes and bone cysts were observed.

An 83-year-old female patient with a body mass index of 22.9, who enjoys golf as a leisure activity, presented with a four-year history of severe pain and deformity in the dorsal region of her right foot. On examination, a bony prominence was observed in the right midfoot, accompanied by tenderness (Figure 1). The Squeeze test was positive, with pain intensifying upon passive plantar flexion of the midfoot. Additionally, a positive piano key sign was noted at the second and third TMT joints. Tapping over the superficial peroneal nerve in this region elicited radiating dysesthesia to the toes. The first TMT instability test was positive, showing a total excursion exceeding 15 mm [4]. The dorsalis pedis pulse was well-palpable. Diagnostic evaluations, including ultrasonography (Figure 2), plain X-ray (Figure 3), and plain CT (Figure 4), identified a dorsal boss in the TMT joint region caused by osteophytes. The patient presents with asymptomatic hallux valgus, with a hallux valgus angle of 28° observed on a weight-bearing X-ray. Preoperative blood tests showed no evidence of rheumatologic markers or signs of inflammation.

Since conservative management had already been attempted at another facility for over four years, surgical intervention was proposed. Dynamic evaluation using ultrasonography revealed joint instability and the potential for increased instability following osteophyte excision was carefully considered (Figure 2).

The operation was carefully planned and carried out following the acquisition of informed consent. An incision was made along the second ray of the toes. Medially, the palpable dorsalis pedis artery was identified. Neurovascular structures overlaying the osteophytes were noted and carefully retracted during the procedure.

**Figure 5:**
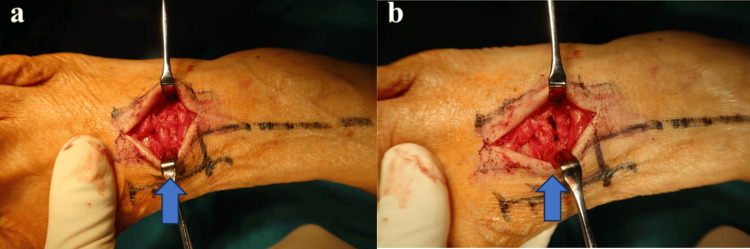
Clinical photographs (a) Neutral position of the midfoot; (b) Plantar flexion position of the midfoot. When the midfoot was plantarflexed, the TMT joint space widened, as indicated by the arrow.

**Figure 6:**
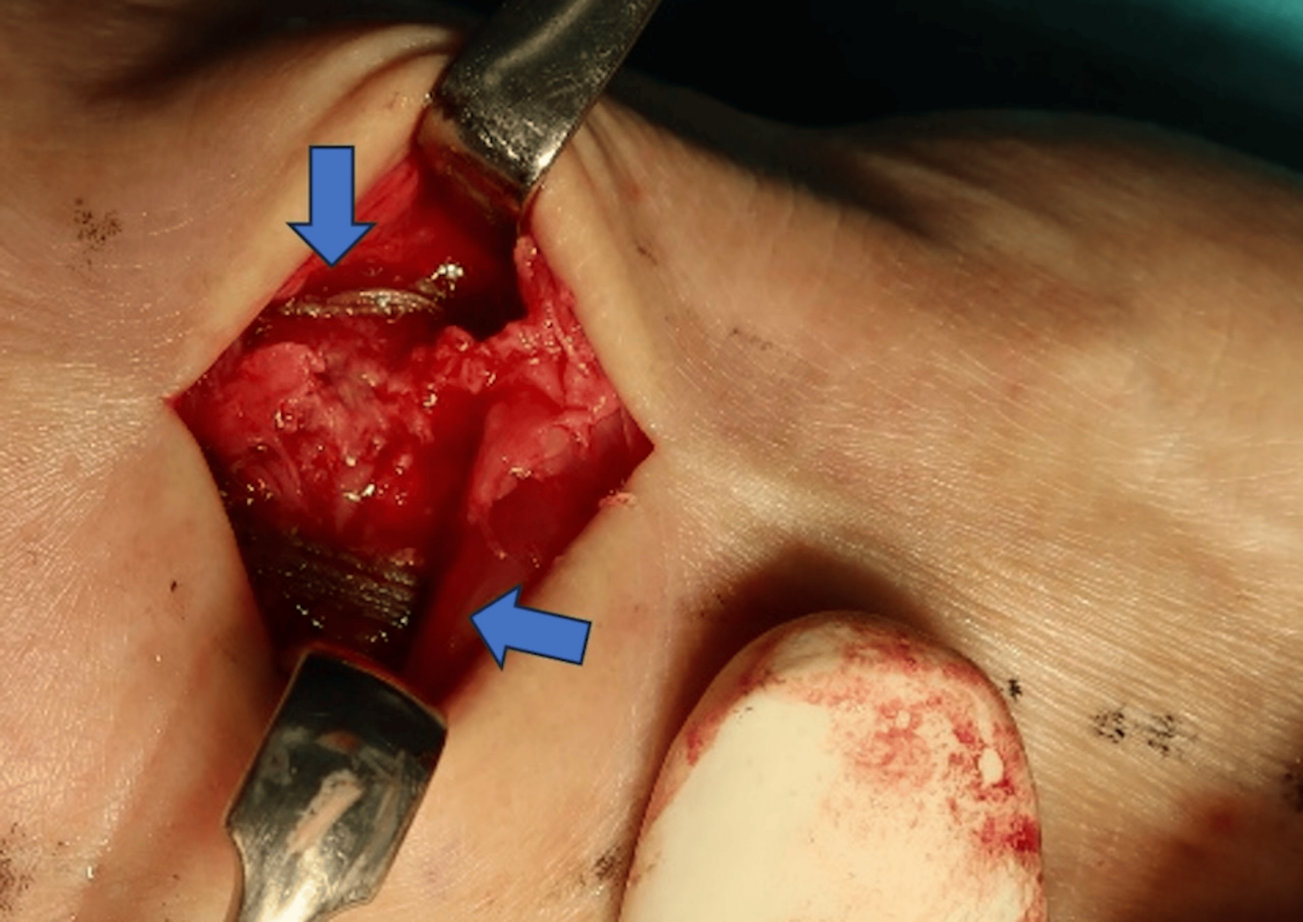
Postoperative clinical photograph The arrows indicate the tape augmentations using the BC Internal Brace (Arthrex, Inc., Florida, USA), which serves to stabilize the joints and prevent the regrowth of dorsal bosses resulting from instability.

The dorsal ligaments were found to be pathologically thinner and appeared non-functional. The inflamed, reddened synovium was extensively excised. Upon exposing the osteophytes, it became clear that the TMT joint naturally widened. Further widening was observed after osteophyte removal with a bone rongeur (Figure 5). The joint surfaces remained intact, were not curetted, and did not require fusion. Bone wax was applied to the resected surfaces to ensure hemostasis and prevent bone regrowth. Osteophytes were excised, and the dorsal aspects of the second metatarsal-intermediate cuneiform, third metatarsal-lateral cuneiform, and first metatarsal-medial cuneiform joints were semi-rigidly stabilized using absorbable screws and ligament tape. This stabilization was achieved with the BC Internal Brace kits. These kits, comprising the Internal Brace, Suture Anchor, BioComposite SwiveLock, and Suture Button Tape (Arthrex, Inc., Florida, USA) (Figure 6), also help prevent the regrowth of dorsal bosses caused by further instability. The same procedure was also performed for the joint between the first metatarsal and the first cuneiform.

Postoperative management included the commencement of ambulation with arch support starting from the third week, which facilitated the patient’s discharge. Full weight-bearing was permitted as tolerated. The surgical outcome was evaluated using the Numerical Rating Scale (NRS) [5] for pain and the Japanese Society for Surgery of the Foot (JSSF) scale, an objective rating system consistent with the American Orthopaedic Foot and Ankle Society (AOFAS) guidelines [6-8]. The preoperative scores were eight out of 10 for the NRS and 41 for the JSSF.

**Figure 7:**
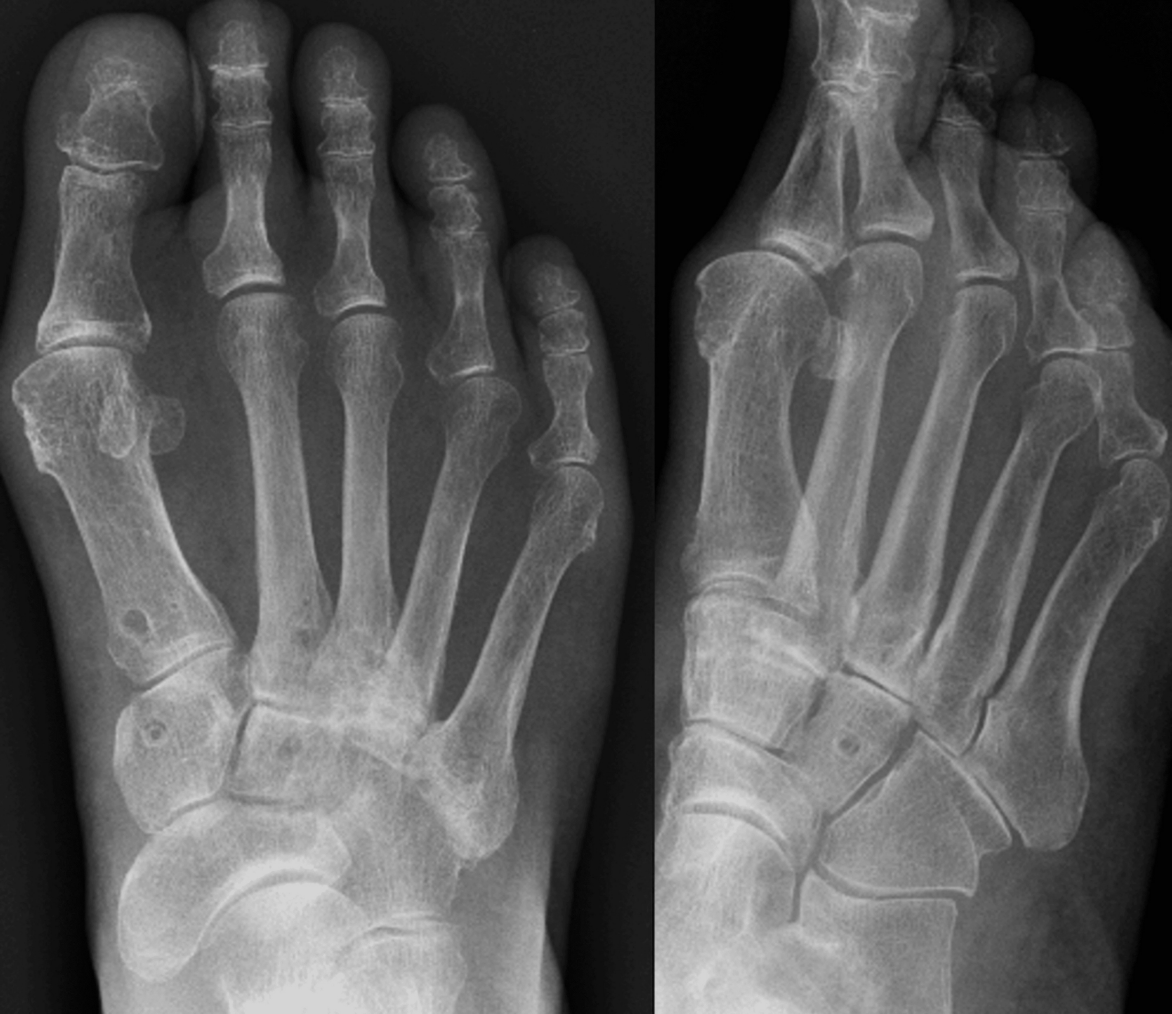
Postoperative X-ray Absorbable screws were carefully inserted into each bone to maintain a moderate tension on the tapes. The fixation of the screw was very secure and stable.

At three weeks post-surgery, the patient had a JSSF score of 67 out of 100. By the fourth week, there was a significant improvement in gait, with the pain score on the NRS score decreasing from eight to five. The patient resumed playing golf three months after the operation. At the final follow-up, two years post-surgery, the patient maintained a JSSF score of 100 and an NRS score of 0. There was no evidence of osteophyte regrowth, and the patient’s golf performance was nearly comparable to pre-surgery levels (Figure 7).

## Discussion

The dorsal boss of the foot, also referred to as tarsal boss, dorsal exostosis, or humped bone, is a bony spur that arises from the intertarsal or TMT joints [9]. Pressure from footwear on the dorsal boss may result in pain, blisters, or corns due to friction. This deformity is sometimes associated with ganglion cysts, adventitious bursitis, and extensor tendonitis [9]. In some cases, the dorsal boss may also impinge on the cutaneous nerve [2,10].

The ligamentous anatomy of the second and third TMT joints is highly intricate and well-structured, with multiple variations classified into six distinct types (Type 1 to Type 6) [11]. Under normal conditions, the second TMT joint exhibits minimal movement, with an arc of only 0.6° in the sagittal plane [12]. In contrast, the third TMT joint demonstrates a range of motion of approximately 4.2°, while the fourth and fifth TMT joints range from 19.2° to 20.7°. These latter joints display significantly greater mobility than the rest of the midfoot, acting as shock absorbers during ambulation on uneven surfaces. Therefore, preserving this mobility is crucial for optimal foot function [12,13].

The literature on the treatment of dorsal boss is limited, and the effectiveness of surgical interventions focused solely on dorsal boss excision, particularly in cases of joint instability, remains uncertain. To date, no studies have definitively established whether resection alone or in combination with fixation offers a superior treatment approach. Bawa et al. reported on a cohort of 26 patients (28 feet) who underwent surgery following failed conservative treatments, with all patients achieving pain relief and returning to full activity within one year [2]. Furthermore, no consensus exists regarding which joints should be stabilized or the extent of stabilization required. Various fixation devices, including Kirschner wires, lag screws, nitinol staples [14,15], and compression plates [16], have been employed in TMT fusion, typically requiring 4-6 weeks of immobilization or offloading to achieve arthrodesis. Additionally, concerns have been raised about the need for hardware removal due to irritation from metal implants [16].

Koroneos et al. compared transarticular screws with a fiber tape construct by measuring diastasis at three Lisfranc joints under various loads. While no significant differences were observed between groups, two failures occurred in the screw group, whereas none occurred in the fiber tape group. This suggests that the fiber tape construct, with a supplemental intercuneiform limb that does not require removal, may offer stability comparable to transarticular screws, even under higher loads [16]. The Arthrex tape ligament, composed of ultra-high-molecular-weight polyethylene (UHMWPE) and an absorbable screw made from 30% biphasic calcium phosphate and 70% poly-L-co-D, L-lactic acid (PLDLA) [17], features a low-profile design that minimizes the risk of skin and nerve irritation.

In our successful case, the range of motion of the second and third TMT joints was found to exceed the reference angle mentioned above [12,13,18], and these joints exhibited signs of instability. The patient was treated with osteophyte excision and semi-rigid fixation as a means of partial anatomical reconstruction of the dorsal segment. The patient presents with mild, asymptomatic hallux valgus. Initially, our team discussed the option of osteotomy for hallux valgus with the patient, as there is literature suggesting that hallux valgus can affect TMT OA [19,20]. However, given that osteotomy requires a minimum of six weeks for union, the procedure was not performed in this case.

The long-term outcomes of tape ligament augmentation remain uncertain. We recognize the potential risk of primary fixation failure of the Internal Brace screw, particularly in cases with bone cysts or severe osteoporosis. When using this device, careful assessment of bone quality via CT scans is essential before surgery. However, in this case, the patient has maintained a perfect JSSF score two years post-surgery, with no signs of instability, regrowth of spurs, or hardware failure. She continues to lead an active and fulfilling life without the need for special orthoses.

In elderly patients, prolonged immobilization or extended hospital stays can lead to premature functional decline, making early reintegration into daily activities essential. In such cases, a semi-rigid, flexible dorsal fixation approach may be preferable to rigid arthrodesis, which typically necessitates extended immobilization and casting. This method is particularly advantageous for dorsal boss excision in the presence of joint instability, as it preserves the joint surface. To our knowledge, this is the first report detailing the treatment of a dorsal boss of the Lisfranc joint using semi-rigid fixation with tape ligament augmentation, showing promising efficacy to date.

## Conclusions

To promote the rapid reintegration of elderly patients into daily activities, we treated the dorsal boss and degenerative TMT joints with osteophyte resection, complemented by semi-rigid fixation using tape ligament augmentation. This technique has shown favorable mid-term outcomes.

At the two-year follow-up, the patient remains pain-free and continues to participate in recreational golf, with no evidence of hardware failure on the X-ray. We consider this procedure a versatile and effective solution, especially in the context of an aging modern society.
